# Supplementary material for: An implantable human stem cell-derived tissue-engineered rostral migratory stream for directed neuronal replacement
Source: Commun Biol. 2021 Jul 15;4:879. doi: 10.1038/s42003-021-02392-8 (PMC8282659; doi:10.1038/s42003-021-02392-8)
Supplement: Supplementary file 2 — Description of Supplementary Files [file 42003_2021_2392_MOESM2_ESM.pdf]

Description of Additional Supplementary Files

File name: Supplementary Data 1

Description: Data summarized in the graphs of Figure 2.

File name: Supplementary Data 2

Description: Data summarized in the graphs of Figure 3.

File name: Supplementary Data 3

Description: Western Blot from Figure 4.

File name: Supplementary Data 4

Description: Data summarized in the graphs of Figure 5.

File name: Supplementary Data 5

Description: Data summarized in the graphs of Figure 6.
