# Supplementary figures and images for: An implantable human stem cell-derived tissue-engineered rostral migratory stream for directed neuronal replacement
Source: Commun Biol. 2021 Jul 15;4:879. doi: 10.1038/s42003-021-02392-8 (PMC8282659; doi:10.1038/s42003-021-02392-8)

## Slide 1
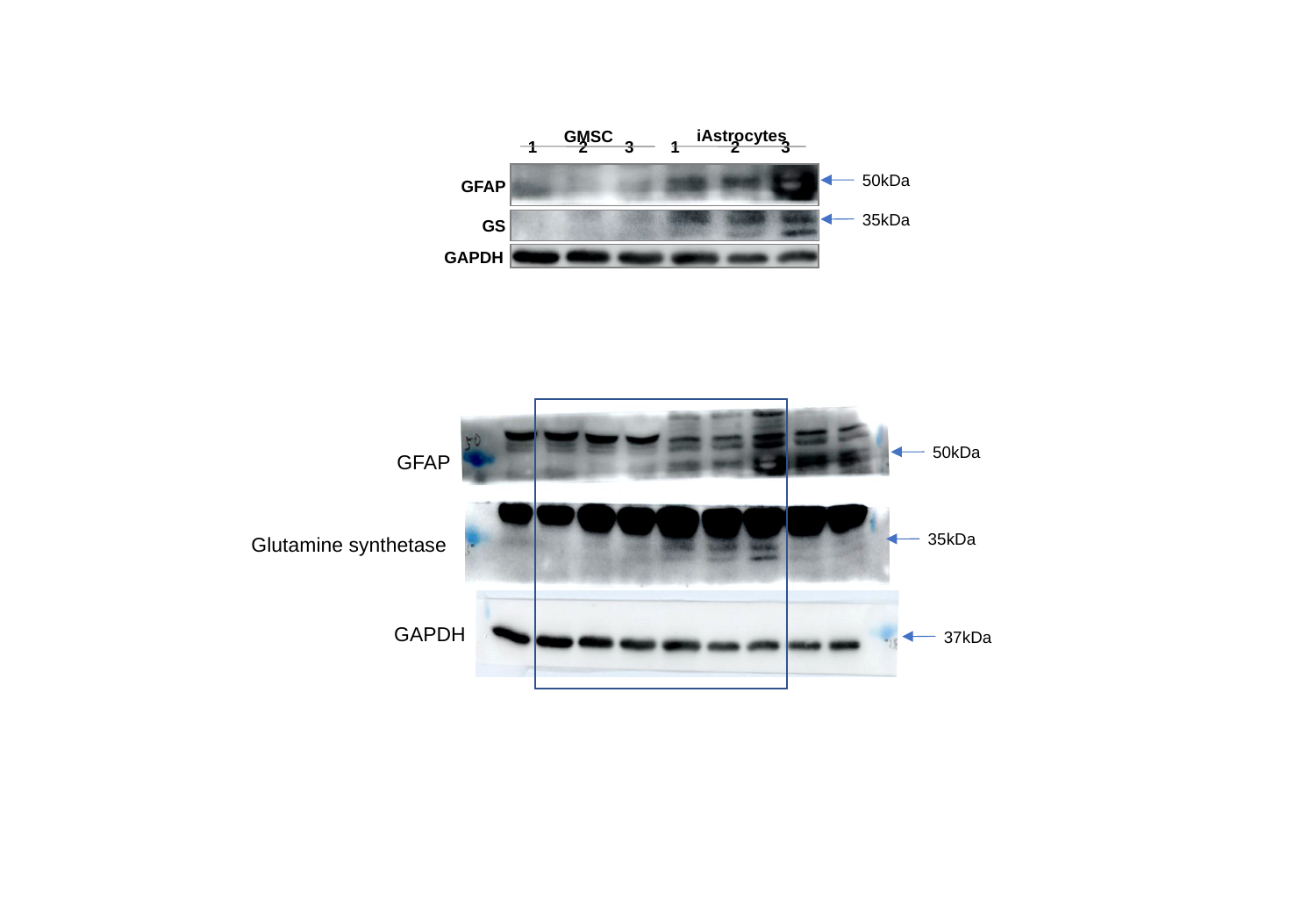

iAstrocytes
GMSC
 1 2 3 1 2 3
50kDa
GFAP
GS
35kDa
GAPDH
50kDa
GFAP
35kDa
Glutamine synthetase
GAPDH
37kDa

Supplement: Supplementary file 5 — Supplementary Data 3 [file 42003_2021_2392_MOESM5_ESM.ppt]
