# Supplementary material for: An implantable human stem cell-derived tissue-engineered rostral migratory stream for directed neuronal replacement
Source: Commun Biol. 2021 Jul 15;4:879. doi: 10.1038/s42003-021-02392-8 (PMC8282659; doi:10.1038/s42003-021-02392-8)
Supplement: Supplementary file 8 — nr-reporting-summary [file 42003_2021_2392_MOESM8_ESM.pdf]

nature research

Corresponding author(s): D. KACY CULLEN

Last updated by author(s): March 31, 2021

Reporting Summary

Nature Research wishes to improve the reproducibility of the work that we publish. This form provides structure for consistency and transparency in reporting. For further information on Nature Research policies, see our Editorial Policies and the Editorial Policy Checklist.

Statistics

For all statistical analyses, confirm that the following items are present in the figure legend, table legend, main text, or Methods section.

n/a	Confirmed	
☐	☒	The exact sample size (*n*) for each experimental group/condition, given as a discrete number and unit of measurement
☐	☒	A statement on whether measurements were taken from distinct samples or whether the same sample was measured repeatedly
☐	☒	The statistical test(s) used AND whether they are one- or two-sided<br>*Only common tests should be described solely by name; describe more complex techniques in the Methods section.*
☒	☐	A description of all covariates tested
☐	☒	A description of any assumptions or corrections, such as tests of normality and adjustment for multiple comparisons
☐	☒	A full description of the statistical parameters including central tendency (e.g. means) or other basic estimates (e.g. regression coefficient) AND variation (e.g. standard deviation) or associated estimates of uncertainty (e.g. confidence intervals)
☐	☒	For null hypothesis testing, the test statistic (e.g. F, t, r) with confidence intervals, effect sizes, degrees of freedom and P value noted<br>*Give P values as exact values whenever suitable.*
☒	☐	For Bayesian analysis, information on the choice of priors and Markov chain Monte Carlo settings
☒	☐	For hierarchical and complex designs, identification of the appropriate level for tests and full reporting of outcomes
☒	☐	Estimates of effect sizes (e.g. Cohen's d, Pearson's r), indicating how they were calculated

Our web collection on statistics for biologists contains articles on many of the points above.

Software and code

Policy information about availability of computer code

Data collection	Nikon-NIS Elements, Fiji
Data analysis	Graphpad Prism

For manuscripts utilizing custom algorithms or software that are central to the research but not yet described in published literature, software must be made available to editors and reviewers. We strongly encourage code deposition in a community repository (e.g. GitHub). See the Nature Research guidelines for submitting code & software for further information.

Data

Policy information about availability of data

All manuscripts must include a data availability statement. This statement should provide the following information, where applicable:

- Accession codes, unique identifiers, or web links for publicly available datasets
- A list of figures that have associated raw data
- A description of any restrictions on data availability

Data supporting the conclusions of this paper are available from the corresponding author upon reasonable request and have been included as supplementary materials with this publication.

Field-specific reporting

Please select the one below that is the best fit for your research. If you are not sure, read the appropriate sections before making your selection.

☒ Life sciences ☐ Behavioural & social sciences ☐ Ecological, evolutionary & environmental sciences

For a reference copy of the document with all sections, see nature.com/documents/nr-reporting-summary-flat.pdf

Life sciences study design

All studies must disclose on these points even when the disclosure is negative.

Sample size	No apriori sample size calculation was performed.
Data exclusions	No data was excluded.
Replication	The experimental findings were reproducible in our laboratory.
Randomization	Animal experiments were limited to a single group, and therefore randomized enrollment was not applicable. Each animal received both control and TE-RMS bilateral implants, thereby controlling for covariates. Likewise, for all TE-RMS constructs fabricated, planar sister cultures were seeded from the same cell suspension for use in in vitro experiments to control for covariates.
Blinding	Blinding was not possible for image analysis due to the clear differences between TE-RMS constructs and planar cultures, and therefore processing and quantification were automated to guard against bias.

Reporting for specific materials, systems and methods

We require information from authors about some types of materials, experimental systems and methods used in many studies. Here, indicate whether each material, system or method listed is relevant to your study. If you are not sure if a list item applies to your research, read the appropriate section before selecting a response.

Materials & experimental systems

n/a	Involved in the study	
☐	☒	Antibodies
☒	☐	Eukaryotic cell lines
☒	☐	Palaeontology and archaeology
☐	☒	Animals and other organisms
☒	☐	Human research participants
☒	☐	Clinical data
☒	☐	Dual use research of concern

Methods

n/a	Involved in the study	
☒	☐	ChIP-seq
☒	☐	Flow cytometry
☒	☐	MRI-based neuroimaging

Antibodies

Antibodies used	Primaries: mouse anti-Ezrin (1:50) (Sigma-Aldrich Cat # E8897, RRID: AB_476955); goat anti-glial fibrillary acidic protein (GFAP) (1:1000) (Abcam Cat # ab53554, RRID: AB_880202); rabbit anti-Robo2 (1:50) (Novus Cat # NBP1-81399, RRID: AB_11013687); mouse anti-CD31 (1:100) (Bio-Rad Cat#: MCA1746GA, RRID: AB_2832958); guinea pig anti-S100B (1:200) (Synaptic systems Cat #: 287 004, RRID: AB_2620025); chicken anti-GFAP (1:1000) (Abcam Cat #: ab4674, RRID: AB_304558); mouse anti-human nuclei (1:200) (Millipore Cat #: MAB1281, RRID: AB_94090); rabbit anti-beta III tubulin (TuJ1) (1:500) (Abcam Cat#: ab18207, RRID: AB_444319); goat anti-doublecortin (DCX) (1:500) (Novus Cat#: NBP1-72042, RRID: AB_11019667); rabbit anti-collagen (1:100) (Abcam Cat#: ab34710, RRID: AB_731684); Hoechst solution (1:10,000) (Invitrogen H3570); rabbit anti-GLAST (EAAT1) (Abcam Cat #: ab41751, RRID: AB_955879); mouse anti-glutamine synthetase (Abcam Cat #: ab64613, RRID: AB_1140869); rabbit anti-laminin (1:500) (Abcam Cat#: ab11575, RRID: AB_298179)<br>Secondaries: donkey anti-mouse 488 (1:500) (Thermo Fisher Scientific Cat#: A-21202, RRID: AB_141607); donkey anti-goat 568 (1:500) (Thermo Fisher Scientific Cat#: A-11057, RRID: AB_2534104); donkey anti-rabbit 647 (1:500) (Thermo Fisher Scientific Cat#: A-31573, RRID: AB_2536183); donkey anti-guinea pig 568 (Sigma Cat#: SAB4600469, RRID: AB_2832959); donkey anti-chicken 647 (Jackson Immunoresearch Cat#: 703-605-155, RRID: AB_2340379); donkey anti-rabbit 568 (Thermo Fisher Scientific Cat #: A10042, RRID: AB_2534017); donkey anti-goat 647 (Thermo Fisher Scientific Cat# A-21447, RRID: AB_2535864);
Validation	All the primary antibodies used were validated for reactivity in rat tissue as stated on the manufacturer's website.

Animals and other organisms

Policy information about studies involving animals; ARRIVE guidelines recommended for reporting animal research

Laboratory animals	astrocyte and neuronal Isolation: Time-pregnant female Sprague Dawley rats<br>Implantation Surgery: Adult male athymic rats (RNU strain 316; Charles River Labs)
Wild animals	Not applicable
Field-collected samples	Not applicable
Ethics oversight	IACUC

Note that full information on the approval of the study protocol must also be provided in the manuscript.
